# Specialist palliative care in different intensive care unit populations: a retrospective longitudinal study

**DOI:** 10.1186/s12904-026-02061-9

**Published:** 2026-03-14

**Authors:** Theresa Tenge, Laura Bosbach, Manuela Schallenburger, Marc Stefaniak, Jacqueline Schwartz, Susanne Feit, Stefan Meier, René M´Pembele, Alexandra Stroda, Sebastian Roth, Jan Gaertner, Rainer Kram, Christian Jung, Detlef Kindgen-Milles, Martin Neukirchen

**Affiliations:** 1 Interdisciplinary Center for Palliative Medicine, Medical Faculty, University Hospital Düsseldorf, Heinrich-Heine-University Düsseldorf, Moorenstraße 5, Düsseldorf, 40225 Germany; 2 Department of Anesthesiology, Medical Faculty, University Hospital Düsseldorf, Heinrich-Heine-University Düsseldorf, Düsseldorf, Germany; 3 Department of Clinical Research, University of Basel, Basel, Switzerland; 4 Palliative Care Center, Bethesda Hospital, Basel, Switzerland; 5 Department of Cardiology, Pulmonology and Vascular Medicine, Medical Faculty, University Hospital Düsseldorf, Heinrich-Heine-University Düsseldorf, Düsseldorf, Germany

**Keywords:** interdisciplinary care, serious illness, end-of-life, palliative medicine, critical care

## Abstract

**Background:**

Integration of specialist palliative care (PC) in intensive care units (ICUs) is recommended but remains underutilized. Understanding differences among ICU populations (i.e., medical vs. surgical) is crucial to overcome barriers and guide optimized care.

**Methods:**

Retrospective longitudinal analysis of surgical, medical-neurological and COVID-19 ICU patients receiving specialist PC consultations at a tertiary care center between 2018 and 2022. Measures and outcomes included patient characteristics, timing of PC involvement, PC triggers according to ICU and PC teams, multi-dimensional symptom assessments and care trajectories.

**Results:**

518 cases were included, 268 (51.7%) from surgical, 174 (33.6%) from medical-neurological and 76 (14.7%) from COVID-19 ICUs. 39.0% of patients (mean age 67 years, 38.6% female) had cancer, pronounced in the surgical population, while medical ICU patients were older and more often female. The first PC encounter was in median 5 (interquartile range [IQR] 1–14) days after ICU admission with an ICU length of stay of 11 (IQR 5–26) days. ICU mortality was 77.2%, hospital mortality 87.6% with 11.6% of all patients were admitted to PC unit. While ICU teams predominantly saw malignancy, lack of curative treatment options and the need to care for next-of-kin as PC triggers, the PC teams mainly the need to care for next-of-kin and symptom burden. In the surgical ICU, malignancy was more often present as a trigger (maximum standardized mean difference [mSMD]: 0.676), for the medical ICU no curative options (mSMD: 0.256) and cardiopulmonary resuscitation (mSMD: 0.375). Symptom assessment regarding pain (mSMD: 0.676), depressive mood (mSMD: 0.552) and dyspnea (mSMD: 0.468) differed between ICU populations. 28.6% of patients had advance directives (*p* = 0.16 between ICUs), 48.1% a power of attorney (*p* = 0.22 between ICUs).

**Conclusions:**

In this pandemic-era cohort, ICU patients receiving specialist PC showed high mortality and symptom burden but differed regarding PC triggers and symptom assessments between ICUs. Further, perceptions of PC triggers and symptoms vary between ICU and PC teams. Future studies in non-pandemic settings are needed to determine the broader applicability of these observations.

**Supplementary Information:**

The online version contains supplementary material available at 10.1186/s12904-026-02061-9.

## Background

As populations age worldwide, the rise in chronic and critical illnesses is reshaping healthcare systems. Simultaneously, invasive treatments such as mechanical ventilation and organ replacement therapies at the end-of-life and the number of deaths occurring in intensive care units (ICUs) are rising to up to 27–43% [1–3]. Given this relevant proportion of patients in life-limiting situations, the integration of palliative care (PC) is recommended but not widely implemented as an essential component of interdisciplinary ICU care [4–6]. According to the European Society of Intensive Care Medicine, PC is a *“specialized medical care for people with a serious illness focused on providing relief from the symptoms and stress of the illness. The goal is to improve quality of life for both the patients and their families”* [7]. PC can be delivered as *general* or *primary* PC by ICU clinicians and nurses and/or *specialist* PC provided by a multi-professional team in a consultative model. It is recommended that both, ICU and PC teams, work together to address the palliative needs of intensive care patients [7, 8]. Evidence supports the benefits of PC in improving care trajectories and addressing physical, psychosocial, and spiritual needs of patients [9–12]. Despite these benefits and an increasing awareness of PC needs in the ICU setting, specialist PC involvement in the ICU remains underutilized and often occurs late in the patient care process as end-of-life care [13–16]. Various trigger factors have been proposed to identify patients who would benefit from specialist PC consultations, with up to 20% of ICU patients meeting these criteria [17]. However, triggers are insufficient, often not universally accepted and have demonstrated poor performance in detecting actual PC needs [18–21]. This highlights the need for a deeper understanding of specialist PC delivery in the ICU to promote established quality indicators [22, 23].

The Coronavirus SARS-CoV-2 (COVID-19) pandemic disrupted healthcare on all levels and led to an increased integration of specialist PC in ICU settings, further emphasizing its importance in co-managing critically ill patients [24, 25]. Previous studies investigated PC in different acute healthcare settings [26, 27]. Data comparing PC delivery in the ICU and medical-surgical wards showed differences in patient demographics, with ICU patients being younger and more likely to have non-cancer diagnoses [27]. ICU patients also tend to have different reasons for PC consultation requests, focusing more on goals of care, comfort care, and withdrawal of interventions and less likely for pain and symptom management. These findings suggest that a comparison of specialist PC delivery in different populations could advise resource allocation and inform educational needs for healthcare providers [27].

Thus, understanding the current state of specialist PC in different ICU populations might help to approach its challenges and guide future care planning for optimizing care in these vulnerable patient populations. Accordingly, the primary aims of this study were descriptive: to characterize and compare (a) triggers for specialist PC consultation, (b) symptom burden, and (c) care trajectories across surgical, medical, and COVID-19 ICUs, and between ICU and specialist PC teams. Although this cohort includes patients admitted during the COVID-19 period, the aim of the study was not to foreground the pandemic but to characterize PC consultation patterns across a diverse ICU patient population. The COVID-19 subgroup is therefore presented as part of the overall case-mix rather than as a defining feature of the analysis. As contemporary ICU practice has moved beyond the acute pandemic era, we reference COVID-19 only insofar as it contributes to understanding variability in consultation timing and triggers, without implying ongoing pandemic-specific relevance.

## Methods

### Study design and ethics

We conducted a retrospective longitudinal study of all specialist PC consultations for adult patients in the ICU from June 2018 to December 2022 at a tertiary care hospital with more than 80 intensive care beds distributed over five ICUs with the following subspecialties: cardiothoracic surgery, general surgery, neurosurgery, neurology, internal medicine. For this study, all surgical ICUs were grouped as “surgical” and internal medicine as well as neurology ICUs as “medical”. During the COVID-19 pandemic, a specialized “COVID-19 ICU” was opened between end 2020 and beginning of 2022. Staffing for the COVID-19 ICU was largely drawn from the surgical ICUs. Access to PC was similar to other ICUs. A long-established specialist PC service at the institution contains an in-hospital eight-bed PC unit with admission criteria based on individual patient needs and bed availably, an inpatient consultation team and outpatient services. Involvement of the inpatient consultation team consists of patients’ contact to specialist PC nurses, to a specialist PC physician, and if needed to other specialist PC team members (e.g., psychological and social support, physiotherapy, creative therapy, and/or spiritual care). Regular multi-professional team discussions about each patient’s needs and treatment goals are performed. No standardized trigger for inpatient specialist PC integration exists at the institution, however, during the COVID-19 pandemic, the team was regularly requested in COVID-19 ICU patients requiring extracorporeal membrane oxygenation (ECMO) [24]. PC requests can be made in the electronic hospital information system, only by physicians. In the surgical ICUs, weekly ward rounds are performed by both, the ICU and PC teams to reflect on potential needs for specialist PC consultations. This process was introduced through the commitment of surgical ICU consultants. For this study, we included all adult ICU patients with a documented specialist PC involvement during their ICU stay. Analyses were based on the level of “PC involvement”. As sensitivity analysis, data were also described on the unique patient level, including only the first involvement per patient. Ethical approval from the local ethics committee was obtained prior to the study start (date of approval: December 2nd 2022, reference number: 2022–2096). Reporting followed the Strengthening the Reporting of Observational Studies in Epidemiology (STROBE) guidelines [28], although some items related to follow-up or time-to-event analyses are not applicable due to the primarily cross-sectional and descriptive nature of our analyses.

### Data collection

Data were collected from the electronic hospital information system used at our institution (CGM MEDICO^®^, Koblenz, Germany). Information regarding the patient’s demographics and characteristics of the ICU admission and specialist PC involvement were obtained. From the consultation requests and specialist PC notes, symptom assessments according to the request form for the ICU and the modified Minimal Documentation System (MIDOS) for the PC teams and the trigger indicating specialist PC integration were collected [29]. Categories for trigger factors were developed based on previous literature, definitions for trigger criteria can be found in Table S1 [18, 30]. The free text-notes and symptom check boxes were grouped into categories by one reviewer not blinded to ICU type. Multiple trigger criteria were possible to apply according to the notes. Absence of documentation for symptoms or triggers was coded as “symptom not present” or “trigger not present”. As outcome parameters, ICU length of stay and discharge location were recorded.

### Data analyses and reporting

This study is a descriptive, exploratory analysis. No formal primary endpoint or hypothesis testing was pre-specified, and all comparisons across ICU populations and between ICU and PC teams are intended to provide descriptive insights rather than test formal hypotheses. Comparisons between ICU and PC teams were based on available documented assessments, which differed between teams and may have influenced observed difference. Continuous variables are described as means with standard deviation (SD) or median with interquartile ranges (IQR), as appropriate. Categorical variables were reported as counts (n) and percentages (%). As this was a hypothesis-generating study, we abstained from defining a primary endpoint. We assessed differences in patient characteristics, PC timing and trajectories between ICU subspecialties using absolute standardized mean differences (SMD). SMD of 0.2, 0.5, and 0.8 are considered small, medium, and large, respectively [31]. Symptom intensities at first and last specialist PC contact ranging from 0 (= none) to 3 (= severe) were visualized using radar plots. Symptom assessments and are planning instruments by the different ICUs and code status before and after specialist PC involvement were compared using chi-square tests with *p* < 0.05 considered as statistically significant. Statistical analyses were performed using STATA (Version SE 16.0, StataCorp LLC, College Station, Texas).

## Results

### Patient characteristics

**Fig. 1:**
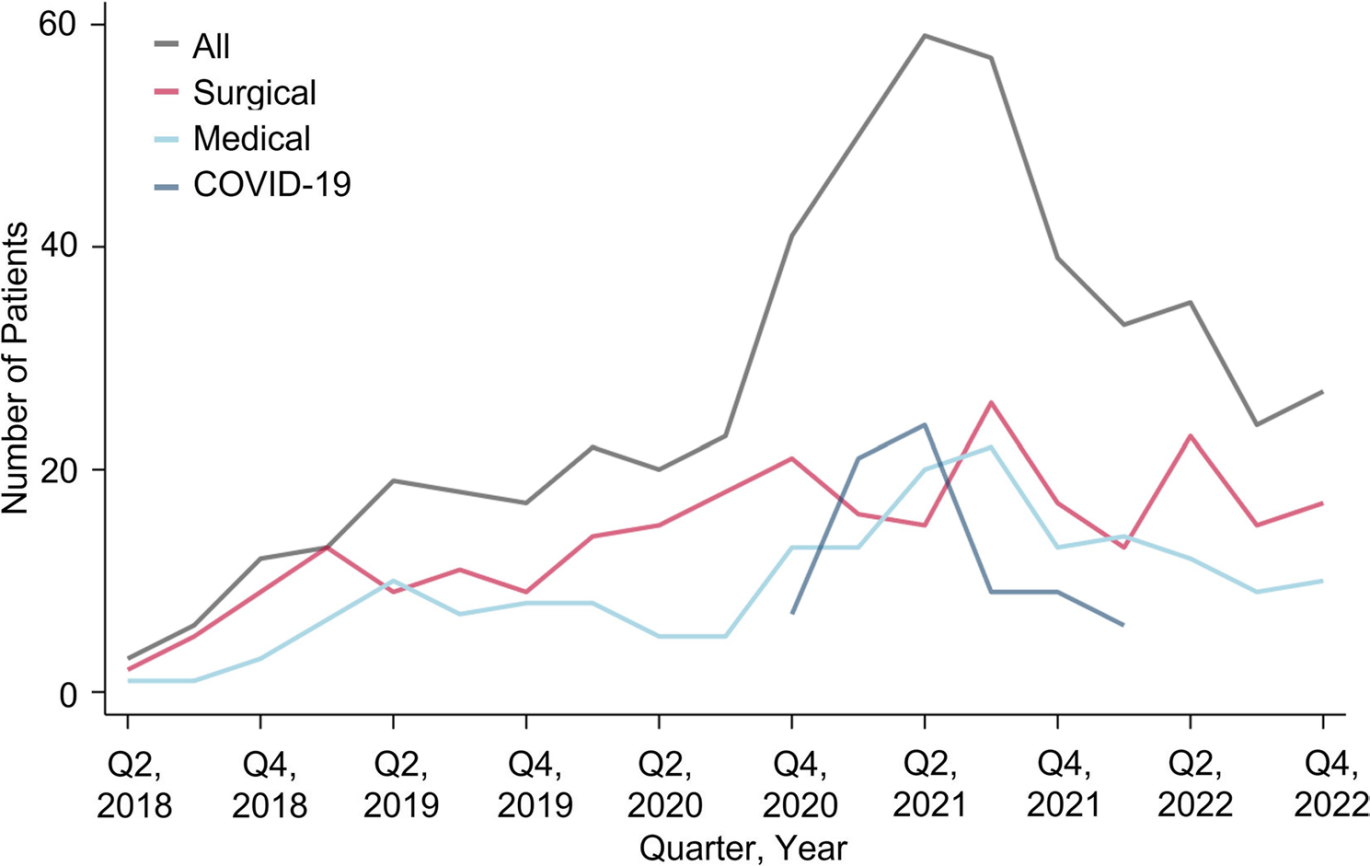
Number of intensive care unit (ICU) patients with specialist palliative care consultation over time in quarterly intervals

Between June 2018 and December 2022, a total of 518 specialist PC involvements during ICU stays for 484 patients with an increasing trend could be obtained (Fig. 1). Among all 518 specialist PC involvements, 268 (51.7%) occurred in surgical, 174 (33.6%) in medical and 76 (14.7%) COVID-19 ICUs. While 202 (39.0%) of all 518 patients had an oncologic disease, in the surgical ICU population almost half of patients suffered from cancer (Table 1).

**Table 1:** Patient characteristics Data are presented as means with standard deviation (SD) or counts (n) and percentages (%).Absolute maximum standardized differences greater 0.2 indicate a considerable difference

	Alln = 518	Medical ICUn = 174	Surgical ICUn = 268	COVID-19 ICUn = 76	Absolute maximum standardized difference
Age, years	66.7 ± 13.8	68.4 ± 12.6	66.8 ± 13.9	62.2 ± 15.3	0.445
Sex, female	200 (38.6%)	73 (42.0%)	103 (38.4%)	24 (31.6%)	0.216
Marital status					0.278
Married/life partner	311 (67.3%)	90 (60.4%)	167 (69.9%)	54 (73.0%)	
Separated/widowed	94 (20.3%)	39 (26.2%)	43 (18.0%)	12 (16.2%)	
Single	57 (12.3%)	20 (13.4%)	29 (12.1%)	8 (10.8%)	
Non-oncologic	316 (61.0%)	109 (62.6%)	142 (53.0%)	65 (85.5%)	0.541
Feeding tube	14 (2.7%)	4 (2.3%)	8 (3.0%)	2 (2.6%)	0.042
Tracheostomy	132 (25.5%)	40 (23.0%)	78 (29.1%)	14 (18.4%)	0.140
NIV	64 (13.4%)	16 (9.6%)	24 (10.1%)	24 (32.0%)	0.573
ETT	166 (32.7%)	41 (24.1%)	85 (32.3%)	40 (53.3%)	0.629

*Abbreviations* *ICU* Intensive Care Unit, *NIV* Non-Invasive Ventilation, *ETT* Endotracheal Tube

### Timing and trajectories

The median hospital length of stay was 21 days (IQR 11–45), the minimum and maximum lengths of stay were 1 and 301 days, respectively. For the ICU stay, the median length of stay was 11 days (IQR 5–26). The first specialist PC consultation occurred in median five days after ICU admission without meaningful differences between ICUs (Table 2). The trajectory analyses revealed that 77.2% of patients (400/518) died in the ICU, while 11.6% (60/518) were transferred to the PC unit, with a median stay of 7 days (IQR 3–12). Among those patients admitted to PC unit, 81.4% died there, 11.9% were discharged home and 6.8% to hospice. 2.1% of all 518 patients were discharged home. Characteristics of all cases versus unique patients (*n* = 484) are presented in Table S2**.**

**Table 2:** Hospital trajectories Data are presented as median with interquartile range or counts (n) and percentages (%)

	**Alln = 518**	**Medical ICUn = 174**	**Surgical ICUn = 268**	**COVID-19 ICUn = 76**	Absolute maximum standardized difference
Hospital LOS, days	21 (11–45)	21 (11–34)	23 (12–56)	19 (8–33)	0.293
ICU LOS, days	11 (5–26)	10 (3–20)	11 (5–28)	17 (7–31)	0.208
Hospital admission until PC, days	12 (5–29)	13 (6–27)	12 (6–33)	6 (2–16)	0.337
ICU admission until PC, days	5 (1–14)	6 (1–14)	5 (1–15)	4 (2–14)	0.021
ICU discharge location					0.725
Died	400 (77.2%)	140 (80.5%)	198 (73.9%)	62 (81.6%)	
Other hospital	42 (8.1%)	8 (4.6%)	20 (7.5%)	14 (18.4%)	
Ward	59 (11.4%)	22 (12.6%)	37 (13.8%)	0 (0.0%)	
Care facility	3 (0.6%)	1 (0.6%)	2 (0.7%)	0 (0.0%)	
Home	4 (0.8%)	2 (1.1%)	2 (0.7%)	0 (0.0%)	
Rehab	3 (0.6%)	0 (0.0%)	3 (1.1%)	0 (0.0%)	
PC unit	6 (1.2%)	1 (0.6%)	5 (1.9%)	0 (0.0%)	
Other	1 (0.2%)	0 (0.0%)	1 (0.4%)	0 (0.0%)	
PC unit	60 (11.6%)	19 (10.9%)	41 (15.3%)	0 (0.0%)	0.495
Hospital discharge location					0.536
Home	11 (2.1%)	5 (2.9%)	6 (2.2%)	0 (0.0%)	
Other hospital	41 (7.9%)	8 (4.6%)	19 (7.1%)	14 (18.4%)	
Care facility	4 (0.8%)	1 (0.6%)	3 (1.1%)	0 (0.0%)	
Hospice	3 (0.6%)	2 (1.1%)	2 (0.7%)	0 (0.0%)	
Died	454 (87.7%)	158 (90.8%)	234 (87.6%)	62 (81.6%)	

*Abbreviations* *ICU*, Intensive Care Unit, *LOS* Length Of Stay* PC *Palliative Care

### Trigger

**Fig. 2:**
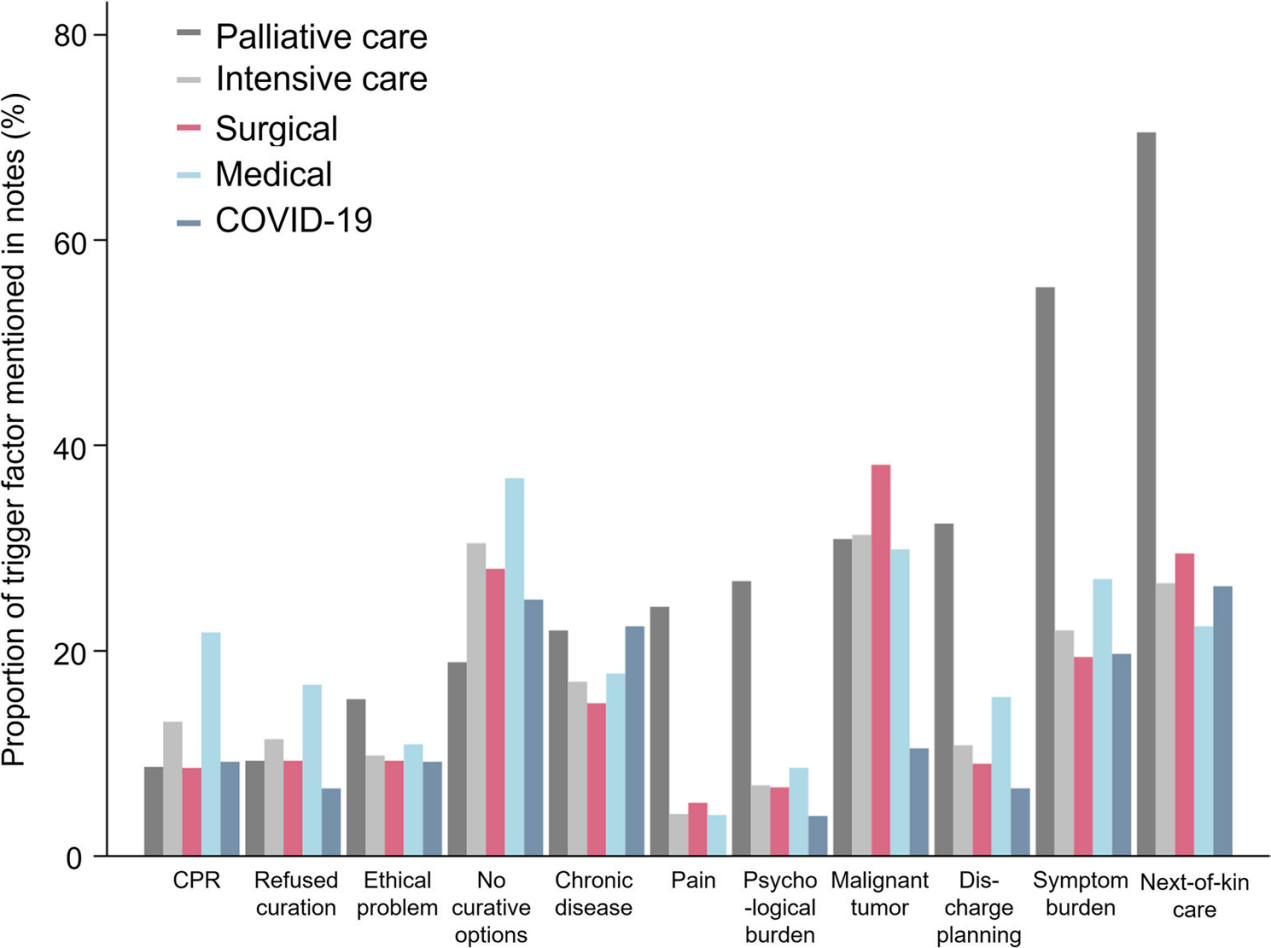
Trigger criteria for palliative care. Proportion of patients with trigger factors frequently mentioned in the notes by ICU and PC teams as well as by different ICU types. Abbreviation: CPR, Cardiopulmonary Resuscitation

The most common triggers for specialist PC consultations in the ICU according to requesting ICU physicians were malignancy (31.3%), lack of curative treatment options (30.5%), and the need to care for next-of-kin (26.6%). While in the medical ICU the most frequent triggers by the ICU physicians were lack of curative treatment options (36.8%), malignancy (29.9%), and symptom burden (27.0%), for surgical patients, malignancy (38.1%), the need to care for next-of-kin (29.5%) and no curative options (28.0%) were most common. In COVID-19 patients, the need to care for next-of-kin was the leading trigger (26.3%), followed by no therapeutic options by the ICU teams (25.0%) and symptom burden (19.7%). The greatest differences for trigger criteria between the ICU populations were seen in malignancy (maximum SMD: 0.676) which was less common in COVID-19 patients, as well as status after cardiopulmonary resuscitation (CPR, maximum SMD: 0.375) and next-of-kin asking for PC (maximum SMD: 0.300) which was pronounced in medical ICUs. Details are provided in Table S3. According to the PC team, patients required specialist PC mainly due to the need to care for next-of-kin (70.5%) as well as symptom burden (55.4%) and discharge planning (32.4%). Overall, notable differences were observed in the documentation of PC triggers between the ICU and PC teams, with more frequent documentation of symptom burden (SMD: 0.729), pain (SMD: 0.607), as well as psychological and spiritual needs (SMD: 0.550 and SMD: 0.285, respectively) by the PC team. Additionally, the trigger discharge planning (SMD: 0.544) and the need to care for next-of-kin (SMD: 0.975) were more frequently documented by PC. Figure 2 summarizes the trigger factors mentioned by ICU physicians in the consultation requests and by PC teams, Table S4 shows the respective data. Table S5 displays trigger criteria of all cases versus unique patients.

### Multi-dimensional assessment

#### Symptom assessment

**Fig. 3:**
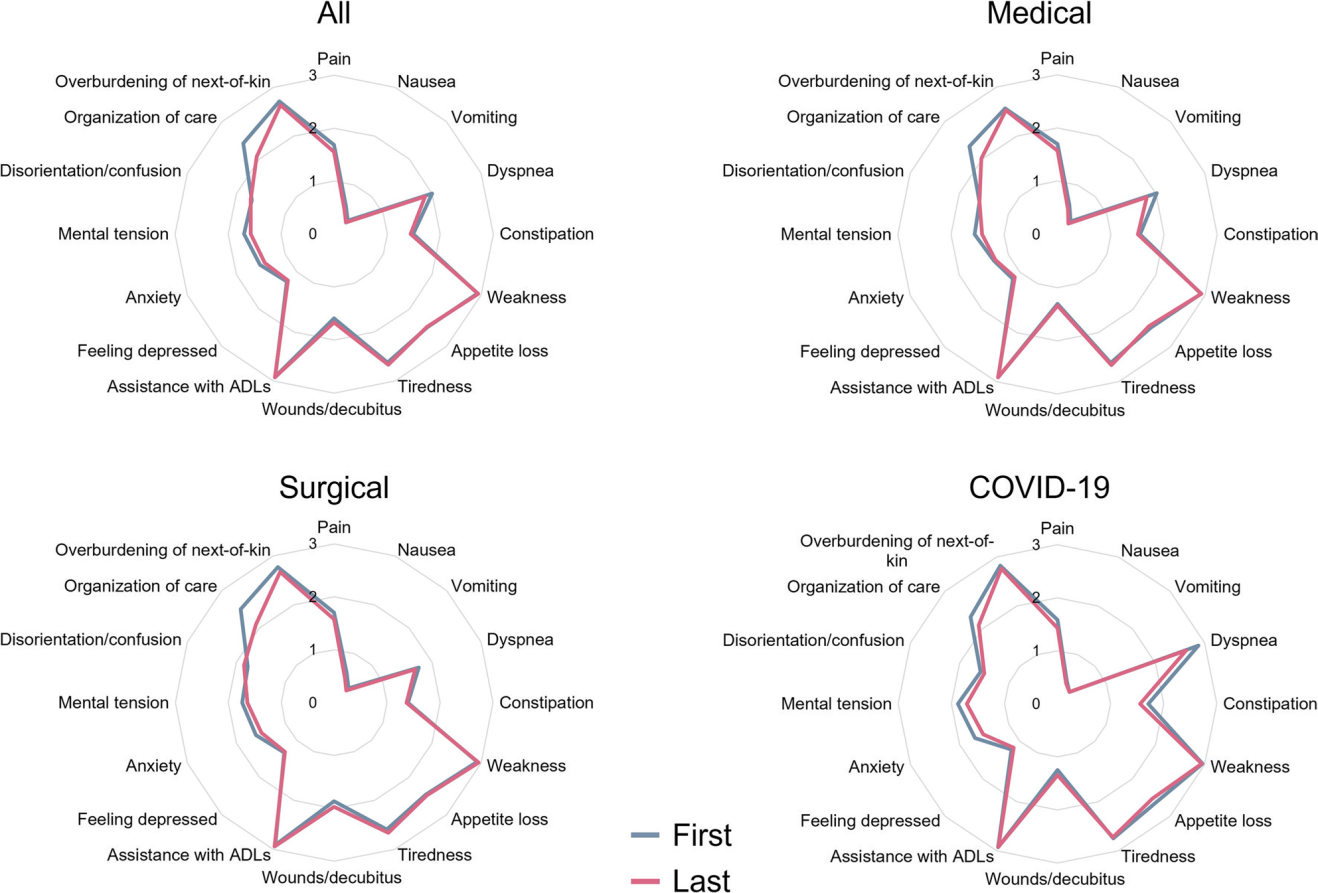
Radar plots for the symptoms in different intensive care populations. Mean symptom intensities ranging from 0 (= none) to 3 (= severe) at first specialist palliative care contact (blue) and last contact (red). ADL, activities of daily living

The most commonly reported symptoms in assessment by ICU teams were weakness/fatigue, overburdening of next-of-kin (substantial emotional or decisional strain among family members), and dyspnea. In the medical ICU group, weakness/fatigue (64.4%) and dyspnea (55.2%) were the most frequently reported symptoms, with overburdening of next-of-kin affecting 50.0%. Anxiety/tension was prevalent in 44.8%, pain in 43.7%, and depressive mood in 36.2% of medical ICU patients. In the surgical ICU, overburdening of next-of-kin (55.6%) and weakness/fatigue (54.9%) were most common, followed by anxiety/mental tension (44.8%), pain (39.2%), and disorientation/confusion (38.1%). There were significant differences in dyspnea (55.2% vs. 31.7%; p < 0.001), depressive mood (36.2% vs. 22.0%; p = 0.001) and disorientation/confusion (27.6% vs. 38.7%; p = 0.023) between medical and surgical ICUs. Among COVID-19 ICU patients, dyspnea (48.7%), overburdening of next-of-kin (43.4%), and anxiety/tension (34.2%) were the most frequent symptoms. Table S6 proved detailed information on each symptom. Differences were observed in symptom assessments between ICU and PC teams documentations. All symptoms were more commonly noted by PC compared to ICU teams with the greatest differences in appetite loss (82.2% versus 30.9%, SMD: -1.230) and wounds and decubitus (66.4.% versus 19.7%, SMD: 1.069), but also in pain (75.3% versus 37.1%, SMD: 0.834), dyspnea (75.1% versus 42.1%, SMD: 0.712) and anxiety (79.5% versus 40.5%, SMD: 0.867). More details are provided in Table S7. Figure 3 illustrates the mean symptom intensities as assessed by the PC teams at first and last contacts. Overall, a relevant decrease in the organization of care burden (SMD: 0.323) and small differences in pain (SMD: 0.137), dyspnea (SMD: 0.135) and mental tension (SMD: 0.123) can be observed (see Table S8). Table S9 presents symptom assessments by the ICU personnel of all cases versus the unique patient level. 

#### Spiritual assessment

A total of 50.8% of patients identified with a religious denomination, including 23.4% Roman Catholic, 11.6% Protestant, and 8.7% Muslim. Chaplain care through the multiprofessional PC team was provided to 1.5% of patients in the ICU, whereas 10.2% of patients in the PC unit received chaplain care.

#### Care planning

Among all 518 patients, 28.6% had an advance directive, 48.1% a power of attorney, and 11.0% a legal guardian. Specific care planning instruments among different ICU populations are displayed in Table 3. The percentage of patients with Do Not Resuscitate (DNR) orders increased from 43.2% (*n* = 224) before consultation to 68.7% (*n* = 356) following PC involvement (*p* < 0.001), while the percentage with Do Not Intubate (DNI) orders rose from 23.2% (*n* = 120) to 47.9% (*n* = 248; *p* < 0.001).

**Table 3:** Care planning instruments

	Medical ICUn = 174	Surgical ICUn = 268	COVID-19 ICUn = 76	*p*-value
Advance directive	50 (28.7%)	83 (31.0%)	15 (19.7%)	0.160
Power of attorney	83 (47.7%)	136 (50.7%)	30 (39.5%)	0.220
Legal guardian	10 (5.7%)	34 (12.7%)	13 (17.1%)	0.074

## Discussion

This study explores characteristics, PC trigger criteria, symptoms and trajectories of patients in surgical, medical and COVID-19 ICUs receiving specialist PC consultations. Different perspectives on the presence of trigger criteria and symptoms by the ICU and PC teams can be observed that allow for reflection of practice during the investigated time window (2018–2022). Our findings collectively highlight several gaps: delayed specialist PC consultation timing, caregiver strain, inconsistent symptom documentation, and minimal spiritual care involvement, underscoring priority areas for strengthening holistic, timely, and needs-based specialist PC integration in the ICU. Importantly, these findings must be interpreted within the unique context of the COVID-19 pandemic, which profoundly altered ICU workflows, referral behaviors, documentation practices, and family involvement.

Of all 518 cases, the surgical ICU accounted for the largest proportion at 51.7%. To provide contextual information, the surgical ICU recorded approximately 3,900 admissions in 2024, with 132 specialist PC consultations, corresponding to an estimated consultation rate of approximately 3%. The medical ICU recorded approximately 1,100 admissions with 65 PC consultations during the same period, which aligns with previous data indicating more frequent specialist PC integration in medical compared to surgical ICUs [30]. Differences in consultation workflows between ICU types with proactive joint-rounds process in the surgical ICUs as well as variations in case mix could influence the observed patterns and should be considered when interpreting the results. Accordingly, statements regarding relative frequency of PC involvement across ICU types are avoided, and these data are presented solely to contextualize the study population rather than to support cross-unit comparisons. As cancer remains the leading primary diagnosis for inpatient specialist PC referral and was more prevalent in the surgical population, this may further help explain our observation, alongside the greater ICU capacity for surgical patients at our institution [32]. Overall, the mean age of 67 years in this cohort is slightly younger than in the general inpatient PC population, which has a mean age of 71 years [32]. However, the COVID-19 population was the youngest, with a mean age of 62 years. While only 15% of patients in the COVID-19 ICU had cancer, nearly half of those in the surgical ICU did. The overall cancer proportion of 39% aligns with previous data [32], though other studies report only 15%, with ICU patients more likely to have non-cancer diagnoses [27]. Recent multicentric data from the United States clearly demonstrated that cancer is a factor promoting specialist PC involvement in the ICU [33].

The first encounter with specialist PC occurred in median five days after ICU admission, consistent with previous studies [14, 30]. Further, the ICU length of stay was comparable [30]. While previous data show that 57% of patients who received specialist PC in the ICU were discharged alive, data from Secunda et al. reported that more than half of these patients died, a trend also reflected in our data, with an ICU mortality rate of 77.2% [30]. Although the specialist PC service is equipped to provide early and comprehensive support for symptom management, communication, and decision-making, our findings indicate that, in this mainly pandemic-era cohort, specialist PC involvement in the ICU were predominantly reactive and focused on end-of-life care, with referrals occurring mainly when patients were already approaching death, as inferred from the observed timing of referrals and high mortality rates, rather than reflecting the intended timing or effectiveness of the service.

It is known that trigger criteria for PC integration vary between populations: ICU patients, compared to normal ward patients receiving inpatient PC, were more likely to have consultation requests for goals of care planning, comfort care, and withdrawal of interventions and less likely for pain and/or symptoms [27]. Also in the general inpatient PC population, advance care planning was the leading reason for referral with 73.5% [32]. In our data, discharge planning was more frequently identified by PC teams, while ICU teams primarily reported malignancy, lack of curative options, and the need to care for next-of-kin as triggers for specialist PC, consistent with previous findings [30]. As prior research has shown differing acceptance of trigger criteria for PC by intensivists and nurses, a deeper understanding of PC need perceptions across professions and disciplines is essential [18, 20].

In addition to trigger assessments, symptom burdens varied between ICU and PC teams. Overall, weakness/fatigue and next-of-kin overburdening were the leading symptoms. Caregiver distress exacerbated by communication barriers, decisional uncertainty, and, during COVID-19, restricted visitation emerged as a prominent trigger for specialist PC involvement, underscoring the crucial role of PC teams in supporting families through emotionally complex decision-making. While the ICU team’s symptom assessment primarily focused on weakness/fatigue and dyspnea, the PC team identified a significant symptom burden in next-of-kin overburdening and anxiety/mental tension. A similar discrepancy in observed symptoms and reasons for consultation was reported by Schoenherr et al., where a substantial percentage of patients had moderate to severe symptoms, yet PC integration was rarely sought for assistance in pain and symptom management [32]. Chapman et al. also reported a high prevalence of symptoms such as pain and dyspnea in ICU patients, with PC team involvement being associated with improvements in both [27]. Our data showed only minimal changes in symptom intensity from the first to the last encounter, which need to be interpreted with caution as this may be caused by documentation practices. Furthermore, we report assessments by the PC teams, which may differ from self-assessment (patient-reported-outcomes). Also, the MIDOS questionnaire is not developed for ICU patients and might not be the optimal to assess symptom burden and PC needs as well as measure effects of specialist PC intervention.

Nearly half of ICU patients had a documented code status, and specialist PC involvement increased the rate of code status completion, consistent with previous findings in PC cohorts [32]. Additionally, other studies have shown that most ICU patients had a full code status at the time of consultation, and that withdrawal of interventions is a common reason for consultation. The authors suggested that specialist PC in the ICU is typically sought when more invasive strategies are no longer effective. This concept of a “time-limited trial” is further supported by the longer length of stay prior to PC consultation in the ICU, which may delay specialist PC involvement [27]. The very limited documentation of spiritual support points to a system-level quality gap and highlights the need for more consistent integration of spiritual care within routine ICU and PC practice [34].

Several limitations come along this single-centric retrospective longitudinal study including the lack of generalizability, limited cohort size and data collected during routine practice. Our institution’s well-resourced specialist PC infrastructure, including an 8-bed PC unit, dedicated consultation team, and weekly joint rounds in surgical ICUs, may not be representative of other centers and could limit the generalizability of our findings to settings with more limited (specialist) PC capacity. Trigger factors were selected based on previous literature and were assigned by a single reviewer who was not blinded to ICU type, and no formal inter-rater reliability assessment or double-coding procedure was performed. This introduces the potential for classification bias. The assignment can further be missed as consultation requests or notes did not mention the trigger although discussed verbally in the team. On the other hand, it can be hypothesized that the written notes focus on what is perceived most important by the team. Both, trigger and symptoms criteria were coded as absent when not documented. This approach carries a risk of misclassification, as lack of documentation does not necessarily indicate absence of symptoms or needs. Additionally, the symptom assessment using MIDOS which gradually ranges symptom intensities from 0 to 3 can be copied from the first assessment using a single button press. Due to time constraints in clinical practice, this may explain the minimal changes observed in symptom intensities over time. Under-documentation and the use of copy-forward in MIDOS and ICU notes may have systematically underestimated or misclassified symptom burden and changes over time, potentially affecting the accuracy of trigger and symptom reporting. Because ICU teams used a simplified yes/no symptom checklist in consultation requests, while the PC team employed the structured MIDOS tool with graded symptom severity ratings, differences in assessment methods and the time devoted to evaluation likely influence reported symptom burden, limiting the direct comparability of ICU and PC team assessments. Analyses were conducted at the level of PC involvements (*n* = 518) rather than unique patients (*n* = 484), resulting in non-independence of observations. No statistical adjustment for within-patient clustering was performed. Consequently, patients with prolonged ICU stays or repeated specialist PC involvement may be overrepresented, potentially inflating the frequency of certain triggers or symptom patterns. To assess the robustness of our findings, we performed a sensitivity analysis restricted to the first PC consultation per patient. The overall distribution of characteristics, consultation triggers and symptom profiles remained qualitatively similar (Supplementary Tables S2, S5, and S9), suggesting that the main conclusions are not solely driven by repeat encounters. Most importantly, this study spans a period of unprecedented deviation from usual ICU workflows, staffing, and referral patterns, and these pandemic-era circumstances represent a major limitation. During this time, triggers for specialist PC consultation were often atypical, visitor restrictions fundamentally altered family needs and communication processes, symptom assessments and documentation were inconsistent, ICU teams were redeployed in nonstandard ways, and the overall ICU case-mix shifted substantially. These factors likely influenced both the frequency and nature of consultations and should temper the interpretation and generalizability of our findings to post-pandemic ICU practice. It is important to note that our dataset is not designed to assess the effectiveness of specialist PC interventions on symptom relief, and the small observed changes in documented symptoms should not be interpreted as indicative of limited specialist PC impact.

## Conclusion

In summary, this study describes patterns of specialist PC consultations across surgical, medical-neurological, and COVID-19 ICU populations including a time of unexpected healthcare disruption by the pandemic. The findings highlight substantial differences in consultation triggers, symptom assessment practices, and the degree of alignment between ICU and PC teams based on available documented assessments. Despite heterogeneity in case-mix, patients were typically referred late in their ICU course, with high mortality and limited completion of advance directives or power-of-attorney documentation, underscoring persistent challenges in the timely identification of PC needs. While part of the cohort reflects pandemic-era conditions with disrupted ICU workflows, case-mix, family presence and documentation practices, the broader patterns, particularly the mismatch between ICU-identified medical triggers and specialist PC-identified communication and symptom needs, highlight ongoing opportunities to strengthen specialist PC integration in the ICU. These findings, limited by pandemic-era influences by the highly specific and atypical period, suggest the need for more consistent symptom assessment, clearer shared referral criteria, and earlier integration of specialist PC across diverse ICU settings, informing efforts to improve the quality of care for seriously ill patients. Future research in non-pandemic settings is necessary to determine broader applicability to contemporary ICU care. 

## Supplementary Information

Supplementary Material 1.

Supplementary Material 2.

## Data Availability

The datasets generated during and/or analyzed during the current study are not publicly available due to data protection but are available from the corresponding author on reasonable request.

## Abbreviations

COVID-19: Coronavirus SARS-CoV-2
CPR: Cardiopulmonary Resuscitation
DNI: Do Not Intubate
DNR: Do Not Resuscitate
ECMO: Extracorporeal membrane oxygenation
ICU: Intensive care unit
IQR: Interquartile range
MIDOS: Minimal Documentation System
PC: Palliative Care
SD: Standard deviation
SMD: Standardized mean differences
STROBE: Strengthening the Reporting of Observational Studies in Epidemiology

## References

[1] Pan H, Shi W. Zhou · Qilong, Chen · Guofeng, Pan · Pengfei. Palliative Care in the Intensive Care Unit: Not Just End-of-life Care. Intensive Care Res 2022. 2022;3(1):1. 10.1007/S44231-022-00009-0.

[2] Karagiannidis C, Krause F, Bentlage C, et al. In-hospital mortality, comorbidities, and costs of one million mechanically ventilated patients in Germany: a nationwide observational study before, during, and after the COVID-19 pandemic. Lancet Reg health Europe. 2024;42. 10.1016/J.LANEPE.2024.100954. 10.1016/j.lanepe.2024.100954 PMC11281923 39070745

[3] Makino J, Fujitani S, Twohig B, Krasnica S, Oropello J. End-of-life considerations in the ICU in Japan: Ethical and legal perspectives. J Intensive Care. 2014;2:1–7. 10.1186/2052-0492-2-9. 25520825 10.1186/2052-0492-2-9 PMC4267582

[4] Michels G, Schallenburger M, Neukirchen M. Recommendations on palliative care aspects in intensive care medicine. Crit Care. 2023;27(1). 10.1186/S13054-023-04622-3. 10.1186/s13054-023-04622-3 PMC10506254 37723595

[5] Neukirchen M, Metaxa V, Schaefer MS. Palliative care in intensive care. Intensive Care Med. 2023;49(12):1538–40. 10.1007/S00134-023-07260-Z. 38010381 10.1007/s00134-023-07260-z PMC10709230

[6] Michels G, John S, Janssens U, et al. Palliative aspects in clinical acute and emergency medicine as well as intensive care medicine: Consensus paper of the DGIIN, DGK, DGP, DGHO, DGfN, DGNI, DGG, DGAI, DGINA and DGPalliativmedizin. Anaesthesiologie. 2023;72(8):590–5. 10.1007/S00101-023-01315-Y. 37394611 10.1007/s00101-023-01315-y

[7] Kesecioglu J, Rusinova K, Alampi D, et al. European Society of Intensive Care Medicine guidelines on end of life and palliative care in the intensive care unit. Intensive Care Med. 2024;50(11). 10.1007/S00134-024-07579-1. 10.1007/s00134-024-07579-1 PMC11541285 39361081

[8] Curtis JR, Higginson IJ, White DB. Integrating palliative care into the ICU: a lasting and developing legacy. Intensive Care Med. 2022;48(7):939–42. 10.1007/S00134-022-06729-7. 35577992 10.1007/s00134-022-06729-7

[9] Aslakson RA, Randall Curtis J, Nelson JE. The changing role of palliative care in the ICU. Crit Care Med. 2014;42(11):2418–28. 10.1097/CCM.0000000000000573. 25167087 10.1097/CCM.0000000000000573 PMC4695994

[10] Doherty C, Feder S, Gillespie-Heyman S, Akgün KM. Easing Suffering for ICU Patients and Their Families: Evidence and Opportunities for Primary and Specialty Palliative Care in the ICU. J Intensive Care Med. 2024;39(8):715–32. 10.1177/08850666231204305. 37822226 10.1177/08850666231204305

[11] Kyeremanteng K, Gagnon LP, Thavorn K, Heyland D, D’Egidio G. The Impact of Palliative Care Consultation in the ICU on Length of Stay: A Systematic Review and Cost Evaluation. J Intensive Care Med. 2018;33(6):346–53. 10.1177/0885066616664329. 27582396 10.1177/0885066616664329

[12] Ito K, George N, Wilson J, Bowman J, Aaronson E, Ouchi K. Primary palliative care recommendations for critical care clinicians. J Intensive Care. 2022;10:1–8. 10.1186/s40560-022-00612-9. 35428371 10.1186/s40560-022-00612-9 PMC9013119

[13] Nadkarni Y, Kukec I, Gruber P, Jhanji S, Droney J. Integrated palliative care: triggers for referral to palliative care in ICU patients. Support Care Cancer. 2022;30(3):2173–81. 10.1007/S00520-021-06542-W. 34704155 10.1007/s00520-021-06542-w

[14] Hsu-Kim C, Friedman T, Gracely E, Gasperino J. Integrating Palliative Care into Critical Care: A Quality Improvement Study. J Intensive Care Med. 2015;30(6):358–64. 10.1177/0885066614523923. 24603677 10.1177/0885066614523923

[15] King D, Schockett E, Rizvi G, et al. The Growth of Palliative Practice and End of Life Care in an Academic Teaching Intensive Care Unit. J Intensive Care Med. 2022;37(10):1397–402. 10.1177/08850666211069031. 35006025 10.1177/08850666211069031

[16] von Saß C, Tenge T, van Oorschot B, et al. Analyzing the use of specialized palliative care in intensive care unit patients in Germany: a cross-sectional study. BMC Palliat Care. 2025;24(1):74. 10.1186/s12904-025-01718-1. 40114097 10.1186/s12904-025-01718-1 PMC11924865

[17] Hua MS, Li G, Blinderman CD, Wunsch H. Estimates of the need for palliative care consultation across united states intensive care units using a trigger-based model. Am J Respir Crit Care Med. 2014;189:428–36. 10.1164/RCCM.201307-1229OC. 24261961 10.1164/rccm.201307-1229OC PMC3977718

[18] Adler K, Schlieper D, Kindgen-Milles D, Meier S, Schallenburger M, Sellmann T, et al. Will your patient benefit from palliative care? A multicenter exploratory survey about the acceptance of trigger factors for palliative care consultations among ICU physicians. Intensive Care Med. 2019;45:125–7. 10.1007/S00134-018-5461-9. 30460500 10.1007/s00134-018-5461-9

[19] Cox CE, Ashana DC, Haines KL, et al. Assessment of Clinical Palliative Care Trigger Status vs Actual Needs Among Critically Ill Patients and Their Family Members. JAMA Netw Open. 2022;5(1). 10.1001/JAMANETWORKOPEN.2021.44093. 10.1001/jamanetworkopen.2021.44093 PMC8777568 35050358

[20] Schallenburger M, Schwartz J, Icks A, et al. Triggers of intensive care patients with palliative care needs from nurses’ perspective: a mixed methods study. Crit Care. 2024;28(1). 10.1186/S13054-024-04969-1. 10.1186/s13054-024-04969-1 PMC11134896 38807236

[21] Igarashi Y, Tanaka Y, Ito K, Miyashita M, Kinoshita S, Kato A, et al. Current status of palliative care delivery and self-reported practice in ICUs in Japan: a nationwide cross-sectional survey of physician directors. J Intensive Care. 2022;10:1–12. 10.1186/s40560-022-00605-8. 35303967 10.1186/s40560-022-00605-8 PMC8932186

[22] Tanaka Y, Masukawa K, Sakuramoto H, Kato A, Ishigami Y, Tatsuno J, et al. Development of quality indicators for palliative care in intensive care units and pilot testing them via electronic medical record review. J Intensive Care. 2024;12:1–15. 10.1186/s40560-023-00713-z. 38195590 10.1186/s40560-023-00713-z PMC10775577

[23] Takaoka Y, Hamatani Y, Shibata T, Oishi S, Utsunomiya A, Kawai F, et al. Quality indicators of palliative care for cardiovascular intensive care. J Intensive Care. 2022;10:1–10. 10.1186/s40560-022-00607-6. 35287745 10.1186/s40560-022-00607-6 PMC8922808

[24] Tenge T, Brimah S, Schlieper D, et al. Specialist Palliative Care Consultations in COVID-19 Patients in the ICU-A Retrospective Analysis of Patient Characteristics and Symptoms at a German University Hospital. J Clin Med. 2022;11(19). 10.3390/JCM11195925. 10.3390/jcm11195925 PMC9571329 36233792

[25] Schoenherr LA, Cook A, Peck S, et al. Proactive Identification of Palliative Care Needs Among Patients With COVID-19 in the ICU. J Pain Symptom Manage. 2020;60(3):e17–21. 10.1016/J.JPAINSYMMAN.2020.06.008. 32544647 10.1016/j.jpainsymman.2020.06.008 PMC7293759

[26] Fassas S, King D, Shay M, Schockett E, Yamane D, Hawkins K. Palliative Medicine and End of Life Care Between Races in an Academic Intensive Care Unit. J Intensive Care Med. 2024;39(3):250–6. 10.1177/08850666231200383. 37674378 10.1177/08850666231200383

[27] Chapman AC, Lin JA, Cobert J, et al. Utilization and Delivery of Specialty Palliative Care in the ICU: Insights from the Palliative Care Quality Network. J Pain Symptom Manage. 2022;63(6):e611–9. 10.1016/J.JPAINSYMMAN.2022.03.011. 35595374 10.1016/j.jpainsymman.2022.03.011 PMC9303815

[28] von Elm E, Altman DG, Egger M, Pocock SJ, Gøtzsche PC, Vandenbroucke JP. The Strengthening the Reporting of Observational Studies in Epidemiology (STROBE) statement: guidelines for reporting observational studies. J Clin Epidemiol. 2008;61(4):344–9. 10.1016/J.JCLINEPI.2007.11.008. 18313558 10.1016/j.jclinepi.2007.11.008

[29] Stiel S, Matthes ME, Bertram L, Ostgathe C, Elsner F, Radbruch L. [Validation of the new version of the minimal documentation system (MIDOS) for patients in palliative care: the German version of the edmonton symptom assessment scale (ESAS)]. Schmerz. 2010;24(6):596–604. 10.1007/S00482-010-0972-5. 20882300 10.1007/s00482-010-0972-5

[30] Secunda KE, Krolikowski KA, Savage MF, Kruser JM. Evaluation of automated specialty palliative care in the intensive care unit: A retrospective cohort study. PLoS ONE. 2021;16(8). 10.1371/JOURNAL.PONE.0255989. 10.1371/journal.pone.0255989 PMC8357176 34379687

[31] Andrade C. Mean Difference, Standardized Mean Difference (SMD), and Their Use in Meta-Analysis: As Simple as It Gets. J Clin Psychiatry. 2020;81(5). 10.4088/JCP.20F13681. 10.4088/JCP.20f13681 32965803

[32] Schoenherr LA, Bischoff KE, Marks AK, O’Riordan DL, Pantilat SZ. Trends in Hospital-Based Specialty Palliative Care in the United States From 2013 to 2017. JAMA Netw Open. 2019;2(12):E1917043. 10.1001/JAMANETWORKOPEN.2019.17043. 31808926 10.1001/jamanetworkopen.2019.17043 PMC6902777

[33] Tenge T, Hadler R, Mahal E, Ahrens E, Wachtendorf LJ, Redaelli S, et al. Development and validation of a prediction score for early identification of palliative care needs for patients in the intensive care unit: a multicentre retrospective cohort study. EClinicalMedicine. 2025;89:103519. 10.1016/J.ECLINM.2025.103519. 41048666 10.1016/j.eclinm.2025.103519 PMC12495436

[34] Choi PJ, Curlin FA, Cox CE. The Patient Is Dying, Please Call the Chaplain: The Activities of Chaplains in One Medical Center’s Intensive Care Units. J Pain Symptom Manage. 2015;50:501–6. 10.1016/J.JPAINSYMMAN.2015.05.003. 26025278 10.1016/j.jpainsymman.2015.05.003 PMC4592806

