# Supplementary material for: Specialist palliative care in different intensive care unit populations: a retrospective longitudinal study
Source: BMC Palliat Care. 2026 Mar 14;25:91. doi: 10.1186/s12904-026-02061-9 (PMC13064263; doi:10.1186/s12904-026-02061-9)
Supplement: Supplementary file 2 — Supplementary Material 2. [file 12904_2026_2061_MOESM2_ESM.docx]

**SUPPLEMENTAL MATERIAL**

**Specialist palliative care in different intensive care unit populations: A retrospective longitudinal study**

**Table S1.** Definitions of trigger criteria

|  | **Definition** |
| --- | --- |
| Patient asks for PC | The patient explicitly expresses a wish for PC involvement. |
| Next-of-kin ask for PC | Family members or close relatives explicitly request PC involvement. |
| No curative options | No curative treatment options are available, and care is limited to life-prolonging or symptom-focused measures. |
| Symptom burden | Presence of severe or refractory physical symptoms (e.g., pain, dyspnea, delirium, anxiety) despite standard ICU management. |
| Malignant tumor | Presence of an active malignant disease with significant impact on prognosis, treatment options, or symptom burden. |
| Meningeosis carcinomatosa | Diagnosis of leptomeningeal carcinomatosis indicating advanced malignant disease with poor prognosis. |
| Refused curation | The patient refuses curative or life-prolonging treatments, opting for comfort-focused or limited interventions. |
| CPR | Cardiac arrest requiring cardiopulmonary resuscitation occurred. |
| ICU > 30 days | Prolonged ICU stay exceeding 30 days. |
| Tracheotomy / PEG after brain injury | Requirement of long-term life-sustaining measures (tracheostomy and/or PEG) following severe brain injury. |
| >3 times ICU readmission | Multiple ICU readmissions (>3). |
| Organ failure (>3 organs) | Failure of more than three organ systems. |
| Intracerebral bleeding and ventilation | Severe intracerebral hemorrhage requiring mechanical ventilation. |
| >80 years and >2 comorbidities | Advanced age combined with multiple comorbid conditions. |
| Brain death | Fulfillment of clinical and legal criteria for brain death. |
| Advance directive | Presence of an advance directive or documented patient wishes relevant to limitation or withdrawal of life-sustaining therapy. |
| Surprise Question | A negative response to the question: “Would you be surprised if this patient died within the next 12 months?” |
| Initial diagnosis not curable carcinoma | Newly diagnosed carcinoma that is incurable at presentation. |
| Conflict within family | Significant disagreement among family members regarding goals of care or treatment decisions. |
| Malignant tumor highly active | Rapidly progressive or treatment-refractory malignant disease with high symptom burden or poor prognosis. |
| Final phase | Clinical signs indicating that the patient is in the last phase of life, with imminent death expected. |
| Apallic syndrome | Persistent vegetative state with no evidence of awareness. |
| Conflict between family and care team | Disagreement between relatives and the medical team regarding prognosis, treatment goals, or ethical decisions. |
| Fasting to death | Voluntary refusal of food and fluids as an expression of end-of-life decision-making. |
| Survival estimation < six months | Estimated life expectancy of less than six months based on clinical judgment. |
| Suicidal | Expression of suicidal ideation, wish to die, or self-harm behavior requiring urgent psychosocial and ethical support. |
| Ethical problem | Presence of ethical dilemmas, such as proportionality of treatment, withholding or withdrawing life-sustaining therapy, or uncertainty about patient wishes. |
| Spiritual needs | Identified spiritual, existential, or religious concerns affecting coping, decision-making, or end-of-life care. |
| Pain | Presence of significant or refractory pain. |
| Discharge planning | Complex discharge planning needs, including transfer home, to hospice, palliative care unit, or long-term care facilities. |
| Patients’ overall situation | Global assessment indicating complex medical, psychosocial, and ethical needs beyond standard ICU care. |
| Psychological burden | Significant psychological distress in the patient, such as anxiety, depression, fear, or delirium. |
| Chronic disease | Presence of advanced or progressive chronic disease contributing to limited prognosis or high symptom burden. |
| Quality of life | Severely impaired or unacceptable quality of life as perceived by the patient or surrogate decision-makers. |
| Pleural effusion/ascites | Recurrent or refractory pleural effusion or ascites. |
| Extensive care | Requirement of highly resource-intensive, invasive, or prolonged life-sustaining therapies. |
| Young patient | Critically ill young patient. |
| Need to care for next-of-kin | The patient’s family or close relatives experience significant emotional, psychological, ethical, spiritual, or decisional burden related to the critical illness that exceeds the primary care team’s competencies and requires additional support. |

**Table S2.** Characteristics of all cases versus unique patients

Data are presented as means with standard deviation (SD) or counts (n) and percentages (%).

Absolute maximum standardized differences greater 0.2 indicate a considerable difference.

|  | **All cases** n = 518 | **Unique patients** n = 484 |
| --- | --- | --- |
| **Age**, years | 66.7 ± 13.8 | 67.0 ± 13.8 |
| **Sex**, female | 200 (38.6%) | 187 (38.6%) |
| **Non-oncologic** | 316 (61.0%) | 296 (61.2%) |
| **Feeding tube** | 14 (2.7%) | 13 (2.7%) |
| **Tracheostomy** | 132 (25.5%) | 119 (24.6%) |
| **NIV** | 64 (13.4%) | 60 (13.3%) |
| **ETT** | 166 (32.7%) | 156 (32.2%) |
| **Hospital LOS**, days | 21 (11–45) | 21 (11–43) |
| **ICU LOS**, days | 11 (5–26) | 11 (5–26) |
| **ICU admission until PC**, days | 5 (1–14) | 5 (1–14) |
| **PC unit** | 60 (11.6%) | 59 (12.2%) |
| **Hospital discharge location** | | |
| Home | 11 (2.1%) | 10 (2.1%) |
| Other hospital | 41 (7.9%) | 40 (8.3%) |
| Care facility | 4 (0.8%) | 4 (0.8%) |
| Hospice | 4 (0.8%) | 4 (0.8%) |
| Died | 454 (87.7%) | 423 (87.4%) |

Abbreviations: ICU, Intensive Care Unit. LOS, Length Of Stay. PC, Palliative Care. NIV, Non-Invasive Ventilation. ETT, Endotracheal Tube

**Table S3.** Trigger criteria by different ICU populations

|  | **All** n = 518  n (%) | **Medical ICU** n = 174  n (%) | **Surgical ICU** n = 268  n (%) | **COVID-19 ICU** n = 76  n (%) | **Absolute maximum standardized mean difference** |
| --- | --- | --- | --- | --- | --- |
| Patient asks for PC | 15 (2.9%) | 7 (4.0%) | 7 (2.6%) | 1 (1.3%) | 0.168 |
| Next-of-kin ask for PC | 37 (7.1%) | 17 (9.8%) | 18 (6.7%) | 2 (2.6%) | 0.300 |
| No curative options | 158 (30.5%) | 64 (36.8%) | 75 (28.0%) | 19 (25%) | 0.256 |
| Symptom burden | 114 (22.0%) | 47 (27.0%) | 52 (19.4%) | 15 (19.7%) | 0.181 |
| Malignant tumor | 162 (31.3%) | 52 (29.9%) | 102 (38.1%) | 8 (10.5%) | 0.676 |
| Meningeosis carcinomatosa | 0 (0.0%) | 0 (0.0%) | 0 (0.0%) | 0 (0.0%) | 0.000 |
| Refused curation | 59 (11.4%) | 29 (16.7%) | 25 (9.3%) | 5 (6.6%) | 0.317 |
| CPR | 68 (13.1%) | 38 (21.8%) | 23 (8.6%) | 7 (9.2%) | 0.375 |
| ICU > 30 days | 17 (3.3%) | 5 (2.9%) | 9 (3.4%) | 3 (3.9%) | 0.059 |
| Tracheotomy / PEG after brain injury | 11 (2.1%) | 4 (2.3%) | 7 (2.6%) | 0 (0%) | 0.231 |
| >3 times ICU readmission | 2 (0.4%) | 1 (0.6%) | 1 (0.4%) | 0 (0%) | 0.107 |
| Organ failure (>3 organs) | 18 (3.5%) | 6 (3.4%) | 9 (3.4%) | 3 (3.9%) | 0.031 |
| Intracerebral bleeding and ventilation | 7 (1.4%) | 1 (0.6%) | 6 (2.2%) | 0 (0%) | 0.214 |
| >80 years and >2 comorbidities | 5 (1.0%) | 2 (1.1%) | 2 (0.7%) | 1 (1.3%) | 0.056 |
| Brain death | 1 (0.2%) | 0 (0.0%) | 1 (0.4%) | 0 (0%) | 0.086 |
| Advance directive | 22 (4.2%) | 10 (5.7%) | 8 (3.0%) | 4 (5.3%) | 0.135 |
| Surprise Question | 0 (0%) | 0 (0.0%) | 0 (0.0%) | 0 (0%) | 0.000 |
| Initial diagnosis not curable carcinoma | 6 (1.2%) | 2 (1.1%) | 4 (1.5%) | 0 (0%) | 0.174 |
| Conflict within family | 5 (1.0%) | 0 (0.0%) | 5 (1.9%) | 0 (0%) | 0.195 |
| Malignant tumor highly active | 2 (0.4%) | 0 (0.0%) | 2 (0.7%) | 0 (0%) | 0.122 |
| Final phase | 12 (2.3%) | 6 (3.4%) | 4 (1.5%) | 2 (2.6%) | 0.126 |
| Apallic syndrome | 1 (0.2%) | 1 (0.6%) | 0 (0.0%) | 0 (0%) | 0.107 |
| Conflict between family and care team | 8 (1.5%) | 1 (0.6%) | 4 (1.5%) | 3 (3.9%) | 0.227 |
| Fasting to death | 0 (0.0%) | 0 (0.0%) | 0 (0.0%) | 0 (0.0%) | 0.000 |
| Survival estimation < six months | 0 (0.0%) | 0 (0.0%) | 0 (0.0%) | 0 (0.0%) | 0.000 |
| Suicidal | 5 (1.0%) | 0 (0.0%) | 4 (1.5%) | 1 (1.3%) | 0.174 |
| Ethical problem | 51 (9.8%) | 19 (10.9%) | 25 (9.3%) | 7 (9.2%) | 0.057 |
| Spiritual needs | 1 (0.2%) | 0 (0.0%) | 1 (0.4%) | 00 (0.0%) | 0.086 |
| Pain | 21 (4.1%) | 7 (4.0%) | 14 (5.2%) | 0 (0.0%) | 0.331 |
| Discharge planning | 56 (10.8%) | 27 (15.5%) | 24 (9.0%) | 5 (6.6%) | 0.287 |
| Patients’ overall situation | 7 (1.4%) | 2 (1.1%) | 3 (1.1%) | 2 (2.6%) | 0.111 |
| Psychological burden | 36 (6.9%) | 15 (8.6%) | 18 (6.7%) | 3 (3.9%) | 0.193 |
| Chronic disease | 88 (17.0%) | 31 (17.8%) | 40 (14.9%) | 17 (22.4%) | 0.191 |
| Quality of life | 0 (0.0%) | 0 (0.0%) | 0 (0.0%) | 0 (0.0%) | 0.000 |
| Pleural effusion/ascites | 11 (2.1%) | 3 (1.7%) | 8 (3.0%) | 0 (0.0%) | 0.248 |
| Extensive care | 1 (0.2%) | 0 (0.0%) | 1 (0.4%) | 0 (0.0%) | 0.086 |
| Young patient | 3 (0.6%) | 0 (0.0%) | 1 (0.4%) | 2 (2.6%) | 0.231 |
| Need to care for next-of-kin | 138 (26.6%) | 39 (22.4%) | 79 (29.5%) | 20 (26.3%) | 0.161 |

**Table S4.** Present trigger criteria by ICU and PC teams

|  | **PC team**  n = 518  n (%) | **ICU team**  n = 518  n (%) | **Absolute standardized mean difference** |
| --- | --- | --- | --- |
| Patient asks for PC | 12 (2.3%) | 15 (2.9%) | 0.036 |
| Next-of-kin ask for PC | 16 (3.1%) | 37 (7.1%) | 0.185 |
| No curative options | 98 (18.9%) | 158 (30.5%) | 0.271 |
| Symptom burden | 287 (55.4%) | 114 (22.0%) | 0.729 |
| Malignant tumor | 160 (30.9%) | 162 (31.3%) | 0.008 |
| Meningeosis carcinomatosa | 1 (0.2%) | 0 (0.0%) | 0.062 |
| Refused curation | 48 (9.3%) | 59 (11.4%) | 0.070 |
| CPR | 45 (8.7%) | 68 (13.1%) | 0.143 |
| ICU > 30 days | 15 (2.9%) | 17 (3.3%) | 0.022 |
| Tracheotomy / PEG after brain injury | 0 (0.0%) | 11 (2.1%) | 0.208 |
| >3 times ICU readmission | 0 (0.0%) | 2 (0.4%) | 0.088 |
| Organ failure (>3 organs) | 18 (3.5%) | 18 (3.5%) | 0.000 |
| Intracerebral bleeding and ventilation | 8 (1.5%) | 7 (1.4%) | 0.016 |
| >80 years and >2 comorbidities | 5 (1.0%) | 5 (1.0%) | 0.000 |
| Brain death | 3 (0.6%) | 1 (0.2%) | 0.062 |
| Advance directive | 23 (4.4%) | 22 (4.2%) | 0.009 |
| Surprise Question | 0 (0.0%) | 0 (0.0%) | 0.000 |
| Initial diagnosis not curable carcinoma | 6 (1.2%) | 6 (1.2%) | 0.000 |
| Conflict within family | 0 (0.0%) | 5 (1.0%) | 0.139 |
| Malignant tumor highly active | 3 (0.6%) | 2 (0.4%) | 0.028 |
| Final phase | 12 (2.3%) | 12 (2.3%) | 0.000 |
| Apallic syndrome | 1 (0.2%) | 1 (0.2%) | 0.000 |
| Conflict between family and care team | 5 (1.0%) | 8 (1.5%) | 0.052 |
| Fasting to death | 0 (0.0%) | 0 (0.0%) | 0.000 |
| Survival estimation < six months | 1 (0.2%) | 0 (0.0%) | 0.062 |
| Suicidal | 6 (1.2%) | 5 (1.0%) | 0.019 |
| Ethical problem | 79 (15.3%) | 51 (9.8%) | 0.164 |
| Spiritual needs | 23 (4.4%) | 1 (0.2%) | 0.285 |
| Pain | 126 (24.3%) | 21 (4.1%) | 0.607 |
| Discharge planning | 168 (32.4%) | 56 (10.8%) | 0.544 |
| Patients’ overall situation | 7 (1.4%) | 7 (1.4%) | 0.000 |
| Psychological burden | 139 (26.8%) | 36 (6.9%) | 0.550 |
| Chronic disease | 114 (22.0%) | 88 (17.0%) | 0.127 |
| Quality of life | 1 (0.2%) | 0 (0.0%) | 0.062 |
| Pleural effusion/ascites | 18 (3.5%) | 11 (2.1%) | 0.082 |
| Extensive care | 0 (0.0%) | 1 (0.2%) | 0.062 |
| Young patient | 2 (0.4%) | 3 (0.6%) | 0.028 |
| Need to care for next-of-kin | 365 (70.5%) | 138 (26.6%) | 0.975 |

**Table S5.** Trigger criteria of all cases versus unique patients

|  | **All** n = 518  n (%) | **Unique patients** n = 484  n (%) |
| --- | --- | --- |
| Patient asks for PC | 15 (2.9%) | 15 (3.1%) |
| Next-of-kin ask for PC | 37 (7.1%) | 37 (7.6%) |
| No curative options | 158 (30.5%) | 150 (31.0%) |
| Symptom burden | 114 (22.0%) | 100 (20.7%) |
| Malignant tumor | 162 (31.3%) | 149 (30.8%) |
| Meningeosis carcinomatosa | 0 (0.0%) | 0 (0.0%) |
| Refused curation | 59 (11.4%) | 55 (11.4%) |
| CPR | 68 (13.1%) | 61 (12.6%) |
| ICU > 30 days | 17 (3.3%) | 17 (3.5%) |
| Tracheotomy / PEG after brain injury | 11 (2.1%) | 11 (2.3%) |
| >3 times ICU readmission | 2 (0.4%) | 2 (0.4%) |
| Organ failure (>3 organs) | 18 (3.5%) | 18 (3.7%) |
| Intracerebral bleeding and ventilation | 7 (1.4%) | 7 (1.5%) |
| >80 years and >2 comorbidities | 5 (1.0%) | 5 (1.0%) |
| Brain death | 1 (0.2%) | 1 (0.2%) |
| Advance directive | 22 (4.2%) | 21 (4.3%) |
| Surprise Question | 0 (0%) | 0 (0%) |
| Initial diagnosis not curable carcinoma | 6 (1.2%) | 6 (1.2%) |
| Conflict within family | 5 (1.0%) | 5 (1.0%) |
| Malignant tumor highly active | 2 (0.4%) | 2 (0.4%) |
| Final phase | 12 (2.3%) | 12 (2.5%) |
| Apallic syndrome | 1 (0.2%) | 1 (0.2%) |
| Conflict between family and care team | 8 (1.5%) | 8 (1.6%) |
| Fasting to death | 0 (0.0%) | 0 (0.0%) |
| Survival estimation < six months | 0 (0.0%) | 0 (0.0%) |
| Suicidal | 5 (1.0%) | 5 (1.0%) |
| Ethical problem | 51 (9.8%) | 46 (9.5%) |
| Spiritual needs | 1 (0.2%) | 1 (0.2%) |
| Pain | 21 (4.1%) | 19 (3.9%) |
| Discharge planning | 56 (10.8%) | 50 (10.3%) |
| Patients’ overall situation | 7 (1.4%) | 7 (1.5%) |
| Psychological burden | 36 (6.9%) | 33 (6.8%) |
| Chronic disease | 88 (17.0%) | 83 (17.2%) |
| Quality of life | 0 (0.0%) | 0 (0.0%) |
| Pleural effusion/ascites | 11 (2.1%) | 10 (2.1%) |
| Extensive care | 1 (0.2%) | 1 (0.2%) |
| Young patient | 3 (0.6%) | 3 (0.6%) |
| Need to care for next-of-kin | 138 (26.6%) | 131 (27.1%) |

**Table S6.** Present symptoms by different ICU populations

|  | **Medical ICU** n = 174  n (%) | **Surgical ICU** n = 268  n (%) | **COVID-19 ICU** n = 76  n (%) | **Absolute maximum standardized mean difference** |
| --- | --- | --- | --- | --- |
| Pain | 76 (43.7%) | 105 (39.2%) | 11 (14.5%) | 0.676 |
| Nausea/vomiting | 30 (17.2%) | 30 (11.2%) | 3 (3.9%) | 0.441 |
| Dyspnea | 96 (55.2%) | 85 (31.7%) | 37 (48.7%) | 0.486 |
| Constipation/diarrhea | 35 (20.1%) | 33 (12.3%) | 4 (5.3%) | 0.456 |
| Weakness/fatigue | 112 (64.4%) | 147 (54.9%) | 25 (32.9%) | 0.452 |
| Appetite loss | 66 (37.9%) | 80 (29.9%) | 14 (18.4%) | 0.442 |
| Wounds/decubitus | 29 (16.7%) | 68 (25.4%) | 5 (6.6%) | 0.529 |
| Depressive mood | 63 (36.2%) | 59 (22.0%) | 10 (13.2%) | 0.552 |
| Anxiety/tension | 78 (44.8%) | 106 (39.6%) | 26 (34.2%) | 0.217 |
| Disorientation/confusion | 48 (27.6%) | 102 (38.1%) | 23 (30.3%) | 0.224 |
| Overburdening of next-of-kin | 87 (50.0%) | 149 (55.6%) | 33 (43.4%) | 0.244 |

**Table S7.** Present symptoms by ICU and PC teams

|  | **PC team**  n = 518  n (%) | **ICU team**  n = 518  n (%) | **Standardized mean difference** |
| --- | --- | --- | --- |
| Pain | 390 (75.3%) | 192 (37.1%) | -0.834 |
| Nausea/vomiting | 199 (38.4%) | 63 (12.2%) | -0.633 |
| Dyspnea | 389 (75.1%) | 218 (42.1%) | -0.712 |
| Constipation/diarrhea | 357 (68.9%) | 72 (13.9%) | -1.345 |
| Weakness/fatigue | 465 (89.8%) | 284 (54.8%) | -0.847 |
| Appetite loss | 429 (82.8%) | 160 (30.9%) | -1.230 |
| Wounds/decubitus | 344 (66.4%) | 102 (19.7%) | -1.069 |
| Depressive mood | 306 (59.1%) | 132 (25.5%) | -0.722 |
| Anxiety/tension | 412 (79.5%) | 210 (40.5%) | -0.867 |
| Disorientation/confusion | 324 (62.5%) | 173 (33.4%) | -0.609 |
| Overburdening of next-of-kin | 440 (84.9%) | 269 (51.9%) | -0.759 |

**Table S8.** Present symptoms at first and last PC contact

|  | **First contact**  n = 518  Mean ± SD | **Last contact**  n = 518  Mean ± SD | **Standardized mean difference** |
| --- | --- | --- | --- |
| Pain | 1.68 ± 0.99 | 1.55 ± 0.95 | 0.137 |
| Nausea | 0.58 ± 0.80 | 0.50 ± 0.72 | 0.103 |
| Vomiting | 0.36 ± 0.70 | 0.31 ± 0.63 | 0.069 |
| Dyspnea | 2.00 ± 1.09 | 1.86 ± 1.06 | 0.135 |
| Constipation | 1.49 ± 0.99 | 1.44 ± 0.98 | 0.051 |
| Weakness | 2.93 ± 0.28 | 2.94 ± 0.26 | -0.064 |
| Appetite loss | 2.49 ± 0.86 | 2.47 ± 0.93 | 0.017 |
| Tiredness | 2.63 ± 0.63 | 2.67 ± 0.61 | -0.071 |
| Wounds/decubitus | 1.59 ± 1.17 | 1.67 ± 1.16 | -0.068 |
| Assistance with ADLs | 2.91 ± 0.34 | 2.93 ± 0.30 | -0.054 |
| Feeling depressed | 1.27 ± 1.07 | 1.23 ± 1.07 | 0.032 |
| Anxiety | 1.51 ± 1.04 | 1.42 ± 1.01 | 0.090 |
| Mental tension | 1.70 ± 1.01 | 1.58 ± 1.01 | 0.123 |
| Disorientation/confusion | 1.68 ± 1.26 | 1.71 ± 1.24 | -0.023 |
| Organization of care | 2.42 ± 0.97 | 2.07 ± 1.20 | 0.323 |
| Overburdening of next-of-kin | 2.71 ± 0.69 | 2.64 ± 0.73 | 0.098 |

**Table S9.** Present symptoms by ICU teams of all cases versus unique patients

|  | **All** n = 518  n (%) | **Unique patients** n = 484  n (%) |
| --- | --- | --- |
| Pain | 192 (37.1%) | 177 (36.6%) |
| Nausea/vomiting | 63 (12.2%) | 55 (11.4%) |
| Dyspnea | 218 (42.1%) | 205 (42.4%) |
| Constipation/diarrhea | 72 (13.9%) | 65 (13.4%) |
| Weakness/fatigue | 284 (54.8%) | 262 (54.1%) |
| Appetite loss | 160 (30.9%) | 149 (30.8%) |
| Wounds/decubitus | 102 (19.7%) | 92 (19.1%) |
| Depressive mood | 132 (25.5%) | 121 (25.0%) |
| Anxiety/tension | 210 (40.5%) | 192 (39.7%) |
| Disorientation/confusion | 173 (33.4%) | 161 (33.3%) |
| Overburdening of next-of-kin | 269 (51.9%) | 254 (52.5%) |
